# Genioplasty with surgical guide using 3D-printing technology: A systematic review

**DOI:** 10.4317/jced.56145

**Published:** 2020-01-01

**Authors:** Olivier Oth, Valérie Durieux, Maria-Fernanda Orellana, Régine Glineur

**Affiliations:** 1 Department of Oral and Maxillofacial Surgery, Hôpital Erasme, Université Libre de Bruxelles (ULB), Route de Lennik 808 1070 Brussels, Belgium; 2 Bibliothèque des Sciences de la Santé, Université Libre de Bruxelles (ULB), Route de Lennik 808 1070 Brussels, Belgium

## Abstract

**Background:**

The purpose of this systematic review is to evaluate the current state of the art of making genioplasties using 3D printing technology.

**Material and Methods:**

A multi-database single-reviewer systematic review identified sixteen papers that fulfilled the selection criteria. There were mainly case series and case reports available (Level IV of the Oxford Evidence-based medicine scale); only two prospective study (Level III) evaluated this subject. These articles are analyzed in details and summarized in this review.

**Results:**

The realization of genioplasties with surgical guide using 3D-printing technology could improve predictability and accuracy. It protects anatomical structures in the environment of the surgery, reducing by this way the morbidity and providing safer results. The type of printer and material used as well as the sterilization techniques should be further developed by the authors. The use of open-access software should also be further explored to allow the use of these new technologies by the largest number of surgeons.

**Conclusions:**

Finally, prospective multi-center studies with larger samples should be performed to definitively conclude the benefits of this new technology and allow for its routine use. This article is the first systematic review on this topic.

** Key words:**Genioplasty, printing, three-dimensional, surgery, computer-assisted.

## Introduction

Genioplasty is a widely used surgical technique used to correct chin deformity. It consists of an osteotomy of the inferior border of the mandible allowing movement of the chin in three dimensions and positioning it in its new desired position. The first surgeon who had performed a chin advancement osteotomy by an extra-oral approach was Otto Hofer (1) on a cadaver. Gillies and Millard (2) applied the same technique on a living patient, also using an external approach. Trauner and Obwegeser (3) were the first surgeons to perform a chin advancement osteotomy via an intraoral approach and called it « genioplasty ». This technique was then modified by several others and used to move the chin in all three dimensions of space: setback genioplasty, impaction genioplasty, vertical height augmentation genioplasty, narrowing genioplasty and widening genioplasty (4).

To obtain the best results in genioplasty, it is essential to make an optimal surgical plan. The osteotomy location and the movement of the bony segment directly impacts the surgical outcome. Traditionally, genioplasty is performed based on surgeon’s intraoperative assessment (5).

In these present times, there is a surge of 3D printing technology in surgical techniques especially in the area of maxillo-facial surgery. 3D printing is also known as rapid prototyping, additive manufacturing and CADCAM technique. These new technologies are revolutionary steps in our way of working as maxillo-facial surgeon (6). Surgical guide is commonly used in dental implantology (7) and its use is now spreading to orthognathic surgery.

The use of a surgical guide obtained with 3D-printing technologies could improve the results of the intervention by a three-dimensional pre-operative simulation and manufacture of per-operative guide(s). This guide / theses guides can aid the surgeon in not touching the surrounding noble anatomical structures (dental roots, alveolar nerve lower) (AKA cutting guide) and / or allow movements of repositioning of the chin in the desired position defined preoperatively (AKA repositioning guide).

The purpose of this systematic review is to evaluate the current state of the art of making genioplasties using 3D printing technology. It will focus mainly on the applications of 3D printing technology currently used in surgically-guided genioplasty and is the first systematic review on this topic.

## Material and Methods

Systematic review of literature

-Literature search 

The systematic literature search was performed in collaboration with a professional research librarian of our university using several well-known databases at the date of 29/10/2018: Medline using PubMed interface and Embase.

The search criteria that were searched for in titles and abstracts relative to the subject of the review were translated into MeSH descriptors, Emtree descriptors and free-text keywords. The precise search equations used in PubMed and Embase are mentioned respectively in Equation 1A and Equation1B (see appendix).

**Figure 1:**
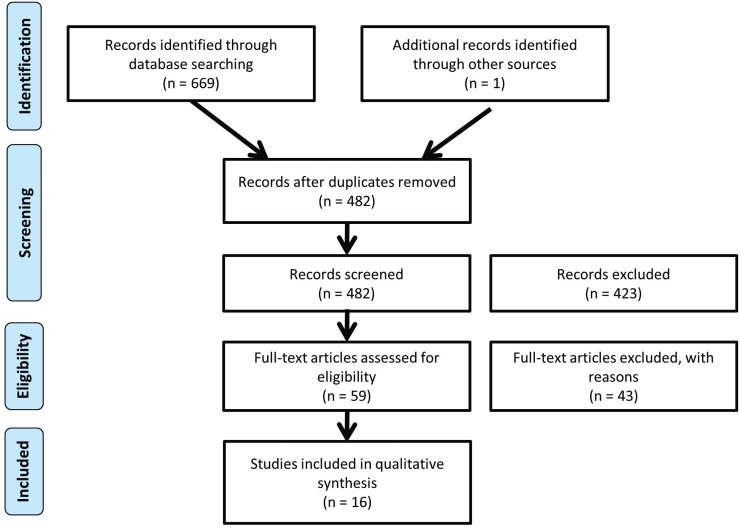
Flowchart.

The PRISMA-flow diagram (Fig. 1) as described by Moher *et al.* (8) , showed the method of our systematic review of literature.

482 title and abstracts were analyzed. The full text version of 59 papers was finally obtained for further analysis.

• Selection criteria 

The following inclusion criteria were chosen to select case series and case reports: 1) Academic publications; 2) publication with the clinical use of a surgical guide obtained by a protocol containing at least one step using 3D printing 3) genioplasty on human patients 4) written in English or French.

•Paper selection 

After analysis by a single reviewer, 15 of the 59 papers fulfilled the selection criteria. The mainly causes of rejection of the article were: off-topic article (use of a surgical guide for Le Fort I osteotomy and/or BSSO osteotomy or mandibular border ostectomy but not for genioplasty, use of chin implants, use of 3D navigation, …), summary of oral presentation or poster, …

To complete the search, the references of each selected publication were searched by hand, only one extra publication following the inclusion criteria was identified.

• Data extraction

The following data were extracted from 16 studies included: type of study; genioplasty alone or associated with other orthognathic techniques; criteria studied to evaluate the technique; number of patients, sex and age; complication(s); results and conclusions of the articles; level of evidence according to the Oxford Center for Evidence-based Medicine. More technical data was also extracted; software(s) used for the surgical simulation, the three-dimensional design and the outcomes evaluation, model of 3D-printer, material used for 3D printing, the type(s) of guide(s) (cutting and/or repositioning), the method of stabilization of the guide.

## Results

The 16 articles selected date from 2010 to 2018 (see  Table 1).

**Table 1:**
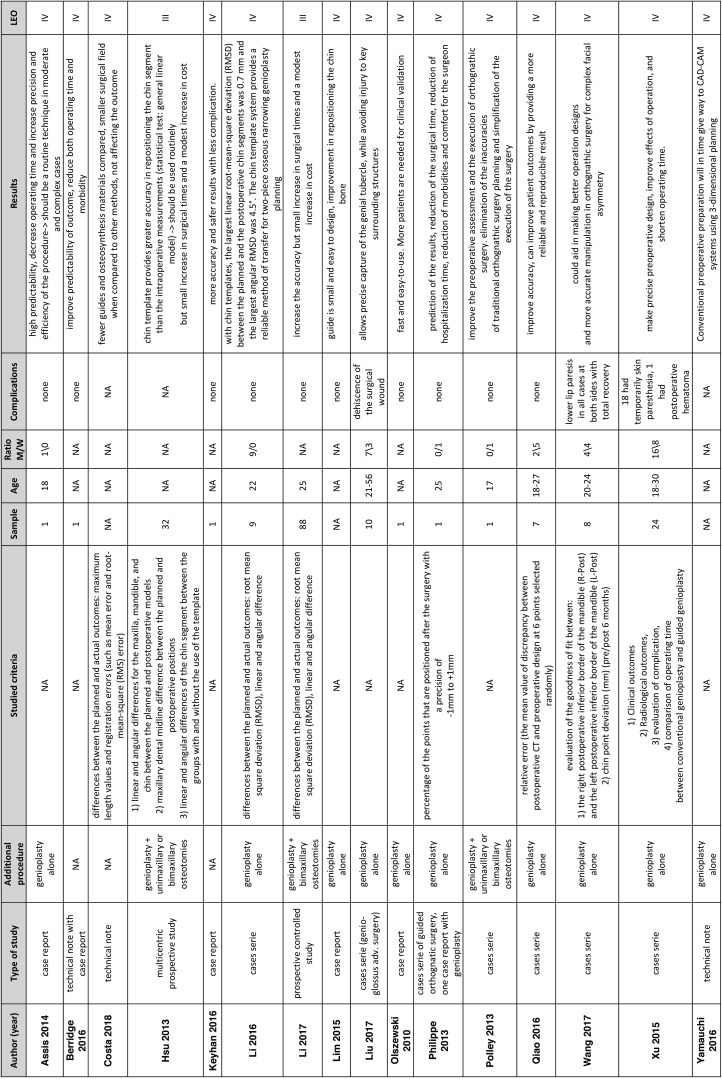
LEO: Level of evidence according to the Oxford Center for Evidence-based Medicine.

All the articles received the agreement of an ethical committee.

Two articles are therapeutic prospective studies (level of evidence type III according to the Oxford Centre for Evidence-Based Medicine 2011 Levels of Evidence) (9): one is multicentric (10) and one is a controlled one (11). The rest of the publications are level of evidence type IV and included cases reports, cases series and technical notes.

In term of associated surgeries, 10 articles evaluate genioplasties alone, 3 associate genioplasties with a single or bimaxillary osteotomy (10-12) and 3 do not communicate information on this subject (13-15).

**Figure 2:**
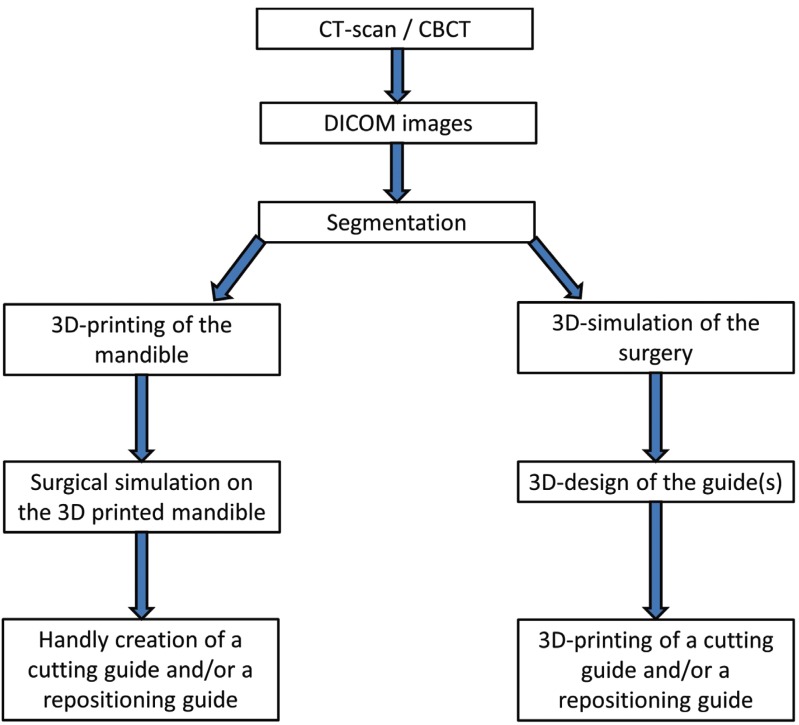
Flowchart.

On the one hand, some of authors use a 3D printed mandible to simulate the surgery and then create a guide on the 3D printed mandible with or without the use of pre-bending plates (13,15-19) ( Table 2, Fig. 2). On the other hand, some authors made a virtually simulated surgery and design virtually one of two guides that are then 3D-printed to be use per-operatively. Theses 3D printed-guides can be used for the osteotomy of the chin (AKA cutting guide)(13,15,20) or for the repositioning of the chin in its desired position after the osteotomy (AKA repositioning guide) (10,14). Some authors used these two type of guides(5,11,12,16,16-19,21-23). With Lim *et al.* (24), one guide is used simultaneous for guiding the cut and then repositioning of the chin.

**Table 2:**
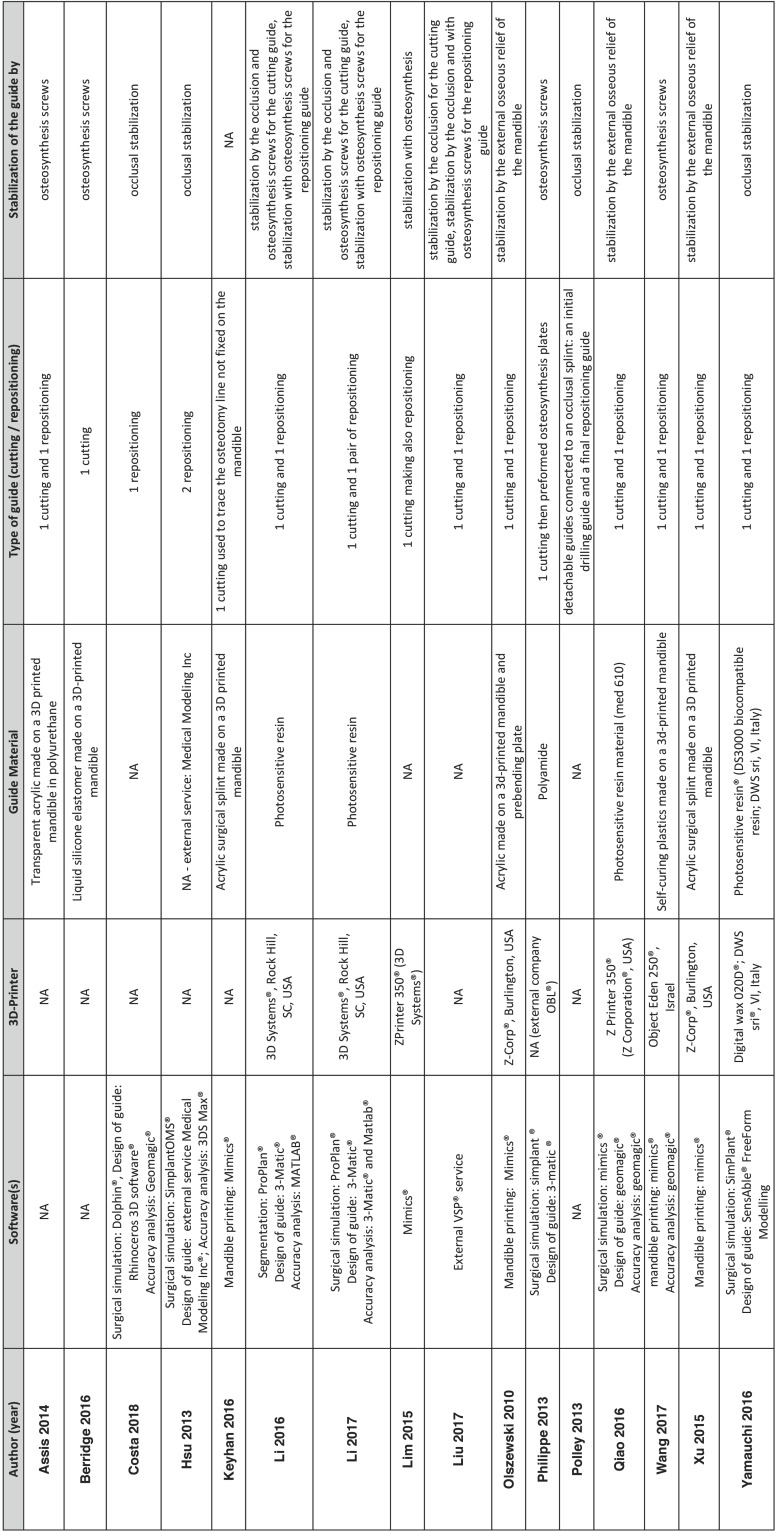
Full softwares reference : Dolphin Imaging software (version 11.5, Chatsworth); Rhinoceros 3D software (version 5.0, Seattle, WA); Geomagic Wrap 2013 software (3D Systems, Rock Hill, SC); Simplant®, Materialise, Belgium; 3DS Max®; Autodesk Inc, San Rafael, CA; MATLAB program (MathWorks, Natick, MA, USA); SensAble® FreeForm Modelling; SensAbleTechnologies Inc, IL, USA; Rapidform XOV2 software®, INUS Technology Inc.

The 3D printing machine was not always mentioned, nor was the 3D printing material. When the guide is created on the 3D printed mandible by hand, it is made of acrylic or elastomer or self-curing plastics; when the guide is 3D printed, it is made of photosensitive resin or polyamide if mentioned.

The softwares used in their protocol are not always mentioned. They can be divided in three type of softwares: 1) software used for the segmentation (= extractions of the zone of interest from the DICOM images created by the scanner of the CBCT) and the surgical simulation (Dolphin®, Simplant OMS®, Proplan®, Mimics®); 2) software used for the design of the guide (Rhinoceros 3D software®, 3-matic®, Geomagic®, SensAble FreeForm Modelling®); 3) software used for the accuracy analysis: Geomagic®, 3DS Max®, Matlab®, 3-matic®).

Guides are stabilized by the external osseous relief of the mandible, or osteosynthesis screws, the occlusion, or by association of these ( Table 2). Two main designs of a genioplasty surgical cutting or repositioning guide brings out these articles: one design of the guide uses the bony variations of the external chin area for the positioning of the guide (17,19,22) ; one other design uses the teeth for positioning of the guide (5,10-12,14,21,23). Half of the authors combine the use of osteosynthesis screws with those techniques for fixing the guide (5,11,13,16,16,18,20,21,24).

The size of the sample varies from 1 to 88 patients. It is summarized in the  Table 1, by age and sex.

8 articles on 15 compare the differences between the planned and actual outcomes, that is to say by comparing the preoperative 3D surgical simulation, thus the position of the chin desired with the surgical simulation and the actual position of the chin segment after the surgery evaluated by a post-operative cone-beam of a post-operative scanner. Philippe (20) studies the percentage of the points that are positioned after the surgery with a precision of -1mm to +1mm. Xu *et al.* (19) have also studied the clinical outcomes and compared the operating time between conventional genioplasty and guided genioplasty

In terms of complications, the only ones described are surgical wound dehiscence, paresthesia of labio-mental fold and one hematoma. These are well-known complications of genioplasty.

Furthermore, as regards of the results and conclusions of the articles, Assis *et al.* (16) and Berridge *et al.* (13) conclude in improvement of predictability ; Assis *et al*. (16), Hsu *et al.* (10), Keyhan *et al*. (15), Li *et al.* (11), Qiao *et al.* (22) and Wang *et al.* (18) conclude in improvement of precision of accuracy. The outcomes and efficiency of the technique are better following Berridge *et al.* (13), Lim *et al.* (24) and Qiao *et al.* (22). Berridge *et al.* (13) and Keyhan *et al.* (15) agree that their techniques reduce the morbidity and give safer results. Costa *et al.* (14) conclude in smaller surgical filed and Polley *et al.*, (12) that it simplifies the execution of the surgery.

Otherwise, Li *et al.* (11) observe a small increase in cost.

Some authors describe a reduction of operating time (13,18), while other describe a small increase of this parameter (5).

Finally, Assis *et al.* (16) and Hsu *et al.* (10) esteem that the technique should be use routinely in moderate and complex cases and following Yamauchi *et al*. (23), 3D printing technologies will in time replace the conventional preoperative preparation.

## Discussion

Assisted-genioplasty using guides issued from 3D-printing technology is really a current topic, indeed the 16 selected articles were published between 2010 and 2018.

The majority of the articles were case report/series with low level of evidence (level of evidence type IV according to the Oxford Centre for Evidence-Based Medicine 2011 Levels of Evidence) (9). The only two prospective studies of level III should be supplemented by others ideally comprising larger samples, being multicentric and controlled to confirm the benefits of these techniques evoked by the different authors. Those benefits seem to affects the surgery (improving of its accuracy and predictability, smaller surgical field, simplification of the technique…), as the patient (protection of the anatomic structures, reduce of morbidity and safer results) and as the society (reduction of the operating time, cost …). All the writers seem to agree on these benefits with the exception of the reduction of time and of cost.

The average duration of a genioplasty from the first incision to the last sutures varies in our experiment from 30 minutes to 90 minutes. This duration may vary according to the experience of the surgeon, the means used to perform the osteotomy (saw vs drill vs. piezotome), and of course the technique of genioplasty used and the bony movements performed (impaction, narrowing in the transverse direction, ...). In this review, only three authors compare the duration of a traditional genioplasty to a genioplasty performed using 3D printing technology with different results: two observe a reduction of the operation time while one describes a small increase of this duration (but without specifying the gain or loss in minutes).

An important parameter that should also be highlighted in addition to the operation duration is the time required for the surgical team to fully acquire these technologies (use of softwares and of the 3D-printer, 3D simulation of the surgery) which also represents a consequent cost. Surgeons indeed use their time to acquire these new technologies instead of operate. The integration of medical engineers in maxillofacial surgery services for the development of 3D-simulation and 3D-printing techniques within the hospital (AKA « in-house techniques ») or subcontracting by external firms are therefore possible alternatives. The use of freeware softwares (computer software available for free on internet) should also be more explored to reduce the costs of theses news technologies. In this review, only one author used a freeware software (23).

Costs will thus vary following the printer, the cost of printing material and the softwares used if the surgeon opts for an in-house 3D laboratory. In our 3D medical laboratory department, the cost for printing material for one genioplasty guide do not exceed 5 euros after the acquisition of a 3D printer and thanks to the use of freeware medical 3D software. Also, the costs for genioplasty guide using external firms range from 400€ to 1000€ in average.

The complications described in the articles are well-known complications of genioplasty, no new complication due to this new technique and no augmentation of the rates of complication are described.

Although the gain in operation time is not clear, and although additional costs are necessary to realize genioplasty with the help of 3D-printing technology, the authors agree on the fact that the comfort of the surgeon is increased, the protection of the anatomical structures is improved and the complications related to the lesion of these anatomical structures are logically diminished. The cost-benefit ratio seems thus largely in favor of the use of a 3D-printing technology during a genioplasty compared to a traditional “blinded” technique. A clinical study was initiated in our department to objectify these different parameters.

Finally, regarding technical details, some authors use an indirect technique to generate guides, but the most accurate seems to be the direct printing of 3D-printed guides. The literature lack of data on the types of 3D printers used and the materials used with these printers to create the guides. This makes the application of these techniques by the maxillofacial community worldwide more difficult. The method of sterilization / disinfection of the guides is poorly mentioned in the literature. It should be systematically mentioned.

## Conclusions

In view of this literature review, the realization of genioplasties with surgical guide using 3D-printing technology seems to be a promising technique that could improve the predictability and accuracy of this surgical technique. It protects anatomical structures in the environment of the surgery, reducing by this way the morbidity and providing safer results. The type of printer and material used as well as the sterilization techniques should be further developed by the authors. The use of open-access software should also be further explored to allow the use of these new technologies by the largest number of surgeons. Finally, prospective multi-center studies with larger samples should be made to definitively conclude on the benefits of this new technology and allow its routine use.
